# Supplementary material for: Multi‐dataset identification of innovative feature genes and molecular mechanisms in keratoconus
Source: J Cell Mol Med. 2024 Sep 19;28(18):e70079. doi: 10.1111/jcmm.70079 (PMC11412914; doi:10.1111/jcmm.70079)
Supplement: Supplementary file 2 — Material S2. [file JCMM-28-e70079-s002.zip › 7.GSEA analysis/2.ATOH7/KEGG_GLYCOSAMINOGLYCAN_BIOSYNTHESIS_KERATAN_SULFATE.html]

Details for gene set KEGG\_GLYCOSAMINOGLYCAN\_BIOSYNTHESIS\_KERATAN\_SULFATE[GSEA]

|  || Dataset | ATOH7.group.cls#h\_versus\_l |
| Phenotype | group.cls#h\_versus\_l |
| Upregulated in class | h |
| GeneSet | KEGG\_GLYCOSAMINOGLYCAN\_BIOSYNTHESIS\_KERATAN\_SULFATE |
| Enrichment Score (ES) | 0.47853124 |
| Normalized Enrichment Score (NES) | 1.3322492 |
| Nominal p-value | 0.17261904 |
| FDR q-value | 0.33098376 |
| FWER p-Value | 0.946 |
Table: GSEA Results Summary

  

Fig 1: Enrichment plot: KEGG\_GLYCOSAMINOGLYCAN\_BIOSYNTHESIS\_KERATAN\_SULFATE      
 Profile of the Running ES Score & Positions of GeneSet Members on the Rank Ordered List

  

| SYMBOL | TITLE | RANK IN GENE LIST | RANK METRIC SCORE | RUNNING ES | CORE ENRICHMENT || 1 | ST3GAL3 | na | 462 | 0.561 | 0.1787 | Yes |
| 2 | FUT8 | na | 1415 | 0.395 | 0.2865 | Yes |
| 3 | ST3GAL2 | na | 2006 | 0.344 | 0.3871 | Yes |
| 4 | CHST1 | na | 3176 | 0.272 | 0.4463 | Yes |
| 5 | CHST4 | na | 4606 | 0.215 | 0.4785 | Yes |
| 6 | B3GNT2 | na | 8386 | 0.110 | 0.4074 | No |
| 7 | B4GALT4 | na | 8571 | 0.107 | 0.4387 | No |
| 8 | B4GALT3 | na | 9016 | 0.099 | 0.4597 | No |
| 9 | B4GALT1 | na | 9610 | 0.088 | 0.4728 | No |
| 10 | B4GAT1 | na | 14793 | 0.023 | 0.3313 | No |
| 11 | B3GNT7 | na | 15627 | 0.016 | 0.3127 | No |
| 12 | B4GALT2 | na | 23726 | -0.066 | 0.1019 | No |
| 13 | ST3GAL1 | na | 23872 | -0.068 | 0.1209 | No |
| 14 | CHST6 | na | 28242 | -0.158 | 0.0490 | No |
| 15 | CHST2 | na | 33383 | -0.403 | 0.0387 | No |
Table: GSEA details [plain text format]

  

Fig 2: KEGG\_GLYCOSAMINOGLYCAN\_BIOSYNTHESIS\_KERATAN\_SULFATE      
 Blue-Pink O' Gram in the Space of the Analyzed GeneSet

  

Fig 3: KEGG\_GLYCOSAMINOGLYCAN\_BIOSYNTHESIS\_KERATAN\_SULFATE: Random ES distribution      
 Gene set null distribution of ES for **KEGG\_GLYCOSAMINOGLYCAN\_BIOSYNTHESIS\_KERATAN\_SULFATE**
